# Supplementary material for: Expression of PDLIM5 Spliceosomes and Regulatory Functions on Myogenesis in Pigs
Source: Cells. 2024 Apr 21;13(8):720. doi: 10.3390/cells13080720 (PMC11049100; doi:10.3390/cells13080720)
Supplement: Supplementary file 1 [file cells-13-00720-s001.zip › cells-2940711-supplementary.pdf]

# Supplementary Information

**Supplementary Table S1. The primer sequences for SqRT-PCR and qPCR**

| Primer name | Primer sequences (5′ to 3′) | Product length (bp) |
|---|---|---|
| *GAPDH* | F: TCGGAGTGAACGGATTTGGC | 189 |
| | R: TGACAAGCTTCCCGTTCTCC | |
| *PDLIM5*-total | F:GCAAAGCATCCCAGGCAAAT | 294 |
| | R: CGCTGTGCGTAGGTATGTGA | |
| *PDLIM5*-short | F: ACATACCTACGCACAGCGAT | 237 |
| | R: CTGAAACTTCATGGTGCCAGTC | |
| *PDLIM5*-long | F: GCTCCTGAATGCGGTCGTT | 224 |
| | R: ATGTCGCCAGCCTCTATGG | |
| *PCNA* | F: GCAGAGCATGGACTCGTCTC | 120 |
| | R: TTGGACATGCTGGTGAGGTT | |
| *CCND1* | F: TTGAAGGCGAGGTTCCAGTC | 163 |
| | R: GCTGGTTCTCTAGGTCAGCC | |
| *CDK4* | F: GGCTACCTCCCGGTATGAAC | 227 |
| | R: CATCCATCAGCCGGACAACA | |
| Ki67 | F: GCCACTTACCTCCATCGCTA | 300 |
| | R: TATAGCGTTTCACTGCCGGG | |
| MyoD | F: TCTAGCAACCCGAATCAGCG | 290 |
| | R: TCGCTGTAATAGGTGCCGTC | |
| MyHC | F: CAGTCTGACGCACCTGAACG | 164 |
| | R: TTTTGCCTCGGTAGCCCTC | |
| MyoG | F: CAGGGGTGCCCAGTGAA | 171 |
| | R: CGTCCTCCACTGTGATGCTG | |

**Supplementary Table S2. The primer sequences for SNP identification of the *PDLIM5* gene**

| Primers | Target regions (bp) | Primer sequences (5′ to 3′) | Amplicon sizes (bp) |
|---|---|---|---|
| P1 | -1927/-110 | F: CCTACGCCATTGTGGTGCT | 1820 |
| | | R: TGAAGCCGTCGCGGAAAAAC | |
| P2 | -1937/-1136 | F: TAACAAGCTGCCTACGCCATT | 801 |
| | | R: TTTCATTGGACGGTGCTAGTCC | |
| P3 | -1359/+86 | F: GAGATTTGTCTCCCAAAGTTACCG | 1439 |
| | | R: CAAGTGACTCCGTTGCGTG | |

**Supplementary Table S3. The primer sequences for promoter region segmentation**

| Primers | Primer sequences (5′ to 3′) | Target regions (bp) |
|---|---|---|
| Promoter1 | F: tctatcgataggtaccCCTACGCCATTGTGGTGCT | -1927/+86 |
| | R: agtaccggaatgccaagcttCAAGTGACTCCGTTGCGTG | |
| Promoter2 | F: tctatcgataggtaccGAGATTTGTCTCCCAAAGTTACCG | -1439/+86 |
| | R: agtaccggaatgccaagcttCAAGTGACTCCGTTGCGTG | |
| Promoter3 | F: tctatcgataggtaccCCAAGTCTTCAAAATCCAGTGTGC | -1054/+86 |
| | R: agtaccggaatgccaagcttCAAGTGACTCCGTTGCGTG | |
| Promoter4 | F: tctatcgataggtaccCTGGAAACTGAACCACTGAGCT | -521/+86 |
| | R: agtaccggaatgccaagcttCAAGTGACTCCGTTGCGTG | |
